# Percutaneous Computed Tomography (CT)-Guided Biopsy of Focal Chest Wall Lesions: A Retrospective Cohort Study

**DOI:** 10.7759/cureus.97985

**Published:** 2025-11-27

**Authors:** Petr Hoffmann, Petr Dvorak, Martin Hyrsl, Martina Hoffmannova, Jindrich Kopecky, Jan Masek, Milan Vajda, Pavel Ryska

**Affiliations:** 1 Radiology, Faculty of Medicine, Charles University, Prague, CZE; 2 Radiology, University Hospital Hradec Kralove, Hradec Kralove, CZE; 3 Faculty of Education, Charles University, Prague, CZE; 4 Oncology and Radiotherapy, Faculty of Medicine, Charles University, Prague, CZE; 5 Oncology and Radiotherapy, University Hospital Hradec Kralove, Hradec Kralove, CZE

**Keywords:** chest wall lesions, complication rate, core needle biopsy, ct guidance, diagnostic accuracy, long-term results, non-vascular intervention, percutaneous approach, procedure safety, therapeutic outcome

## Abstract

Background: This study aimed to retrospectively evaluate the efficiency, accuracy, technical features, and relationships among the monitored parameters of CT-guided percutaneous biopsies of focal chest wall lesions.

Methodology: A total of 143 percutaneous biopsy procedures for tumors, ranging in size from 15 to 168 mm (median size 43 mm), were performed in patients with focal chest wall lesions over 12 years. Only local anesthesia using trimecaine was sufficient for the procedures. The duration of the intervention never exceeded 20 minutes. Needles 14 G, 16 G, or 18 G were used for the biopsies. Histological results were extended with subtyping in the vast majority of cases. In 91 interventions (63.6%), malignant disease was present in the patients´ medical history.

Results: Diagnostic accuracy was achieved in 137 (95.8%) biopsies, while six (4.2%) procedures yielded false-negative histological results later confirmed by surgical excision. Metastatic disease of diverse origins represented the most frequent histological diagnosis (46.2%). Hematologic malignancies were revealed in 29.4% of procedures. Primary tumors were verified in only 13 (9.1%) cases. Complications occurred in 3 (2.1%) procedures, all consisting of minor hemorrhage managed conservatively.

Conclusion: CT-guided percutaneous core needle biopsy of focal chest wall lesions demonstrated high diagnostic accuracy with a minimal incidence of complications.

## Introduction

The chest wall provides protection to the vital organs of the thoracic cavity and comprises skin, fatty and connective tissue, vessels, nerves, cartilage, muscles and bones (ribs, sternum and vertebrae). It forms a dynamic structure that is essential for respiration. Tumors of the chest wall are uncommon lesions that represent less than 5% of all thoracic malignancies [1]. About 50% of focal lesions are benign processes [2]. Final histological results vary widely according to the affected tissue. Simple cysts, hematomas, hemangiomas, lymphangiomas, hamartomas, granulomas, elastofibromas, fibrous dysplasias, desmoid tumors, lipomas, leiomyomas, neurofibromas, chondromas, and osteochondromas represent a large heterogeneous group of benign chest wall lesions [2,3]. Additionally, many rare neoplasms of diverse origin can be verified, including primary malignancies, especially sarcomas [4,5]. Metastatic tumors are one of the most common types of chest wall tumors. Lung and breast carcinomas are the most common malignancies in this group [6]. 

Differentiating lesions as primary vs. secondary and benign vs. malignant is essential to ensure appropriate treatment in each case. Tumors often appear as palpable masses, but more than 20% of processes are asymptomatic and are diagnosed incidentally on different imaging examinations [3]. Various imaging techniques can be employed for detection and detailed characterization. Chest radiography generally serves as the first method of choice. Ultrasound (US) can be used in the management of palpable soft tissue masses; in the establishment of a diagnosis, and as guidance for biopsy [7]. Computed tomography (CT), alone or fused with positron emission tomography (PET/CT), is the gold standard for determining tumor location, differentiating lesion tissue, and tumor extent and can also be used for biopsy navigation [8,9]. Magnetic resonance imaging (MRI), often combined with positron emission tomography (PET/MRI), can be used in select cases [10]. Some focal lesions do not present with typical imaging patterns for various reasons, e.g., previous therapy for other diseases or diffuse chest wall soft tissue infiltration. Additionally, multiple histologically distinct malignancies in the patients´ medical history or previous imaging examinations frequently cause hesitation in diagnosis.

Imaging features alone are not sufficient to establish a definitive diagnosis. Small lesions (less than 5 cm) are usually treated with surgical excision, whereas larger processes are often referred for biopsy [2]. Image-guided biopsy using a percutaneous approach is an established method in these situations [11]. This article presents our long-term experience from over a decade. We aim to confirm the clinical usefulness of the biopsy procedure, emphasize relevant methodological features, and assess its applicability in clinical practice.

## Materials and methods

Over a 12-year period (April 2012-April 2024), 143 patients were retrospectively evaluated. Biopsy indications were established on the basis of clinical and imaging examinations. Chest radiography and/or ultrasound were performed as initial diagnostic steps. In all cases, the initial suspicion was verified through intravenous contrast-medium enhanced CT, 18F-fluoro-2-deoxyglucose (18F-FDG) PET/CT, or MRI in specific cases.

The monitored parameters included patient age, gender, largest lesion diameter, biopsy needle gauge, number of samples obtained, patient’s clinical history of tumors, complications and their consequent management, final histological diagnosis (including subtyping), results of excision biopsy or resection in parallel with the percutaneous approach, and correlations with relevant therapeutic outcomes.

The interventional procedures were performed at different anatomical sites of the chest wall. Supine, prone or lateral positions on the CT table were used as appropriate in all cases. All interventions were performed under CT guidance. A Siemens Somatom Force or Somatom Definition AS Plus (Siemens, Forchheim, Germany) was used. Neither automatic navigation system guidance nor CT fluoroscopy was used at any time.

Fully informed consent was obtained in all cases by the physician performing the biopsy or the treating physician with an explanation of the biopsy course, possible complications and their management, and potential outcomes. All the patients were approved by the multidisciplinary council (thoracic surgeon, radiologist, oncologist, or pulmonologist). This study was approved by the institutional ethics review committee.

Local anesthesia (Trimecaine, Zentiva, Prague, Czech Republic) was sufficient in the procedures performed; neither conscious sedation nor general anesthesia was ever needed. Coagulation parameters, activated partial thromboplastin time (aPTT, lower than 1.5) and International Normalized Ratio (INR, lower than 1.5) were routinely assessed before the biopsy.

The procedure was planned based on the aforementioned diagnostic examinations. The safest and shortest needle approach to reach the lesion was determined. The needle track was planned to avoid the vessels (especially the internal thoracic artery in anterior chest wall biopsies), lungs and pleural cavity, and nerves (particularly in posterior paravertebral procedures). The entry point was defined by placing a mark on the skin. The angle and length of the biopsy needle were measured based on intraprocedural CT imaging with a local anesthesia needle. This brief imaging step is essential for correct needle placement and the localization of the lesion and surrounding organs. The procedures were performed using the Core semi-automatic biopsy system (Palium biopsy, MDL SRL, Delebio, Italy). The needle entry site and surrounding skin were disinfected and covered with sterile drapes. Depending on the chosen approach, a 14 Gauge (G), 16 G, or 18 G needle (10 cm length, 15 or 22 mm throw) was inserted into the lesion. After obtaining the specimen, the needle was carefully extracted from the targeted area. The samples were placed into a sterile 4% formaldehyde solution and transported for further examination. A single-pass biopsy technique was used in all cases.

After the procedure, a limited series of CT scans was performed, covering the same anatomical ranges as in the pre-procedural examination, to exclude potential early complications. The needle puncture site was disinfected and dressed with a sterile plaster. Following biopsy, patients were admitted to the hospital department of pneumology, oncology, internal medicine, or thoracic surgery for monitoring vital functions. The following day, patients were discharged from the departments after undergoing X-rays, clinical, and laboratory examinations.

In patients who subsequently underwent surgical treatment, histological results obtained from resection specimens were compared with those from the biopsy. Concordance between these findings was regarded as diagnostic success. In non-surgically treated patients (e.g. lymphomas, disseminated malignancies, or benign histological results), histopathological results were compared with patients’ prior oncological history and the outcomes of targeted or conservative therapies. For imaging follow-up, CT (same devices as above), PET/CT Discovery VCT 64 (General Electric Healthcare, Milwaukee, Wisconsin, USA) or MRI (Siemens Magnetom Avanto 1.5 Tesla, Siemens, Forchheim, Germany) was used as appropriate.

Histopathological evaluation was performed by board-certified pathologists according to standard diagnostic criteria. Subtyping and additional immunohistochemical or molecular analyses were performed only when clinically indicated.

Retrospective data collection formed the basis of the study. Median and interval data were used for the basic quantitative statistical evaluation. These data were correlated with qualitative parameters using Fisher’s exact test. Qualitative statistical data were descriptively evaluated and compared with quantitative parameters using the nonparametric Mann-Whitney test; for comparisons across more than two groups, the Kruskal-Wallis H test was applied. Statistical significance was established at p = 0.05. Analyses were performed using the statistical software NCSS 11 (NCSS, LLC, Kaysville, Utah, USA).

## Results

A total of 143 interventional procedures with accessible histological results were included in the study. Biopsies were performed for suspected chest wall malignancy in tumors ranging from 15 to 168 mm in maximum diameter (median 43 mm). Of the patients, 85 (59.4%) were men and 58 (40.6%) were women, with ages ranging from 31 to 93 years (median 71 years). Overall, six (4.2%) histological results were considered false negatives. In the remaining 137 (95.8%), biopsy results were regarded as true positives or true negatives. No rebiopsies were performed. The total duration of the intervention never exceeded 20 minutes. In 91 (63.6%) interventions, malignant disease was recorded in the patients’ medical history.

**Figure 1:**
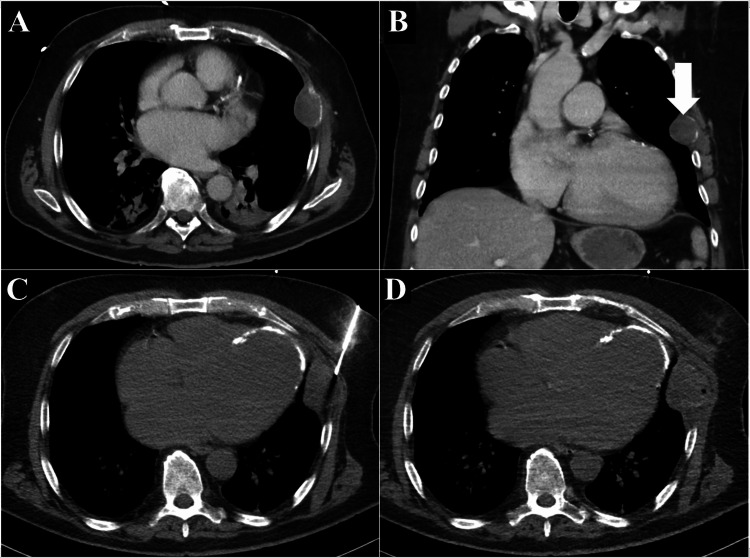
Primary tumor. Hypovascular focal lesion destroying part of the left rib on contrast-enhanced CT in the portal venous phase, axial (A) and coronal planes (B, arrow). The placement of the biopsy needle (C, axial plane, patient in supine position). Postprocedural control computed tomography axial scan demonstrated no complications (D). Definitive histological diagnosis was epithelioid hemangiosarcoma.

Histologically, the most frequent diagnosis was NSCLC, identified in 30 (21%) cases. Primary chest wall tumors were confirmed in 13 (9.1%) cases (Figure 1), metastases in 66 (46.1%) procedures, and lymphoma in 42 (29.4%) interventions. No malignant cells were found in 22 (15.4%) interventions. Table 1 shows a detailed overview.

**Table 1:** The number of verified diagnoses. NSCLC: Non-Small Cell Lung Carcinoma; SCLC: Small Cell Lung Carcinoma; RCC: Renal Cell Carcinoma; DLBCL: Diffuse Large B-Cell Lymphoma; CLL/SLL: Chronic Lymphocytic Leukemia/Small Lymphocytic Lymphoma; T-NHL: T-cell Non-Hodgkin Lymphoma; EBV: Epstein-Barr Virus

	Diagnosis	Number
Primary	Neurinoma	2 (1.4%)
	Epitheloid hemangiosarcoma	2 (1.4%)
	Angiolipoma	1 (0.7%)
	Myoepithelioma	1 (0.7%)
	Angiosarcoma	1 (0.7%)
	Chondrosarcoma	1 (0.7%)
	Synovial sarcoma	1 (0.7%)
	Myeloid sarcoma	1 (0.7%)
	Dedifferentiated sarcoma	1 (0.7%)
	Sarcomatoid carcinoma	1 (0.7%)
	Spinocellular carcinoma	1 (0.7%)
Metastatic	NSCLC	30 (21%)
	Breast carcinoma	12 (8.6%)
	RCC	4 (2.8%)
	Hepatocellular carcinoma	2 (1.4%)
	SCLC	2 (1.4%)
	Upper gastrointestinal tract carcinoma	2 (1.4%)
	Colorectal carcinoma	2 (1.4%)
	Pancreatic carcinoma	2 (1.4%)
	Cholangiocarcinoma	1 (0.7%)
	Urothelial carcinoma	1 (0.7%)
	Melanoma	1 (0.7%)
	Esophageal carcinoma	1 (0.7%)
	Prostate carcinoma	1 (0.7%)
	Thymic carcinoma	1 (0.7%)
	Laryngeal carcinoma	1 (0.7%)
	Parotid carcinoma	1 (0.7%)
	Tonsillar carcinoma	1 (0.7%)
	Neuroendocrine tumor	1 (0.7%)
Hematology	DLBCL	17 (11.9%)
	Plasmacytoma	11 (7.8%)
	CLL/SLL	3 (2.1%)
	T-NHL	2 (1.4%)
	Mantle cell lymphoma	2 (1.4%)
	Marginal zone lymphoma	2 (1.4%)
	Follicular lymphoma	1 (0.7%)
	Hodgkin lymphoma	1 (0.7%)
	Plasmablastic lymphoma	1 (0.7%)
	Chronic myeloid leukemia	1 (0.7%)
	EBV-associated lymphoproliferative disease	1 (0.7%)
	No tumorous cells verified (incl. false negative)	22 (15%)
Number		143 (100%)

For preprocedural diagnosis, contrast-enhanced CT was used in 83 (58%) cases, MRI in 19 (28.7%) cases, and PET/CT in 41 (13.3%) cases. CT was most often performed for establishing an initial diagnosis due to its wide availability. PET/CT was optimal in patients with suspected recurrence of a known malignancy, lymphoma relapse or transformation, while MRI was preferred in younger patients and in cases involving the dorsal chest wall, thoracic spine, or nerve structures. Statistical data analysis showed no significant relationship between biopsy accuracy and the preprocedural imaging modality (p = 0.856).

A 16 G needle was optimal in 126 (88.1%) biopsies, an 18 G needle in 13 (9.1%) interventions, and a 14 G needle in four (2.8%) procedures. The 16 G gauge was adequate for most biopsies. The thinner 18 G needle was reserved for lesions near large vessels, typically in the parasternal space along the internal thoracic artery. The larger 14 G needle was used in bulky heterogeneous masses when extended histological subtyping was expected and a larger tissue sample was required. Fisher’s exact test showed no significant relationship between needle gauge and diagnostic accuracy (p = 0.487). Skin-to-lesion distance was not a relevant parameter for measurement, due to the superficial location of the vast majority of cases.

**Figure 2:**
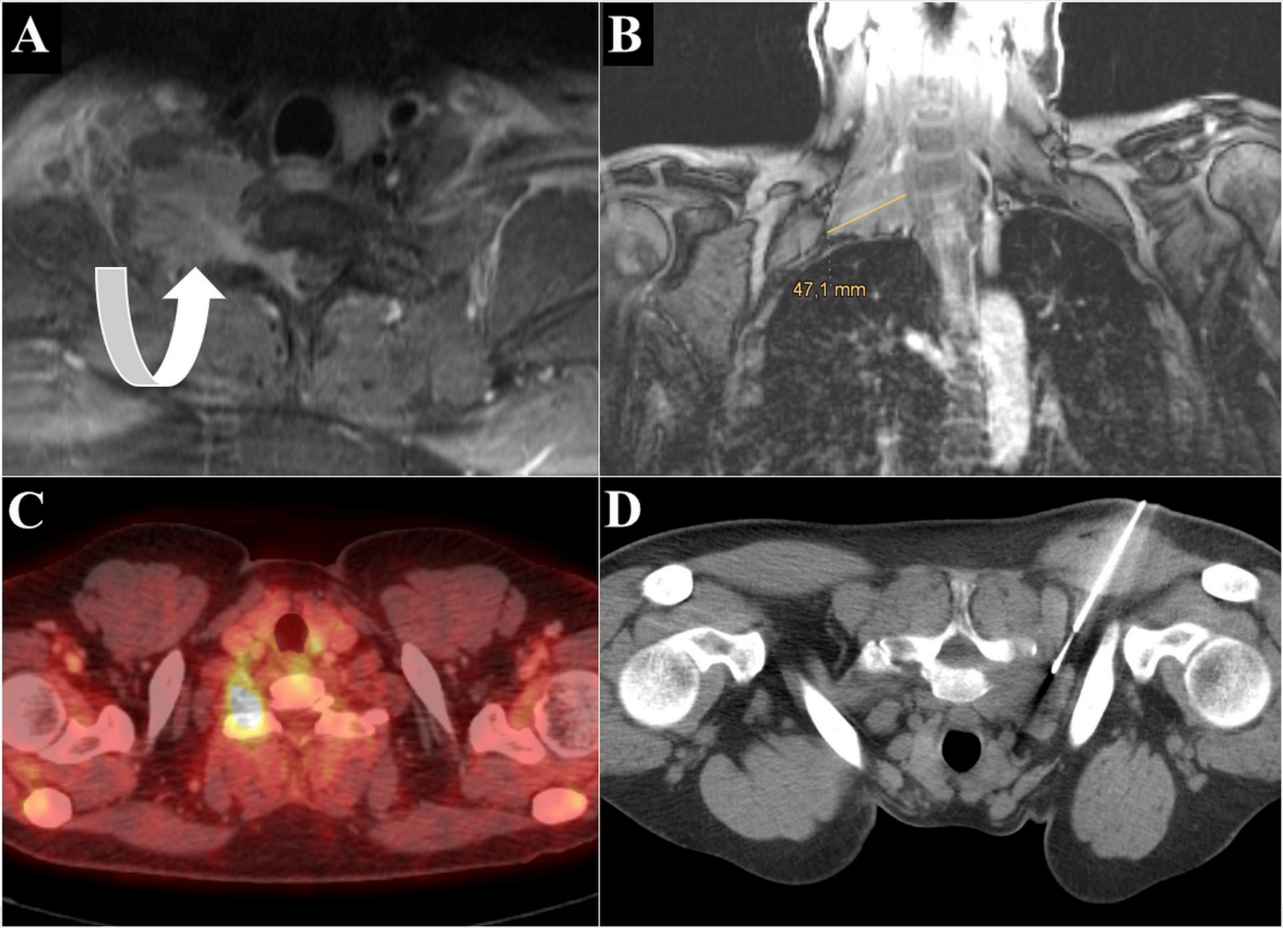
Apical tumor biopsy. Vascular lesion in the apex of the right chest wall invading the first rib and vertebra, shown on an MR T1 sequence in transverse (A, curved arrow) and coronal (B) planes, and on positron emission tomography/computed tomography (C). Intraprocedural CT showing biopsy needle placement (D, prone position). Definitive histological diagnosis was diffuse large B-cell lymphoma (DLBCL).

The number of biopsy attempts varied: One attempt was performed in eight (5.6%) interventions, two samplings in 69 (48.3%) biopsies, three samplings in 51 (35.6%) cases, four samplings in 11 (7.7%) cases, and five samplings in four (2.8%) biopsies were used. A single sampling was sufficient for high-risk interventions near large arteries in the parasternal space or at the apical thoracic wall (Figure 2). Two or more samples were sufficient in 135 (94.4%) biopsies, with two attempts performed in the majority of procedures. More than three samplings were optimal for large masses, with an emphasis on targeting multiple regions of the lesion. Fisher’s exact test showed no significant relationship between the number of biopsy attempts and histological results, including subtyping (p = 0.515).

**Figure 3:**
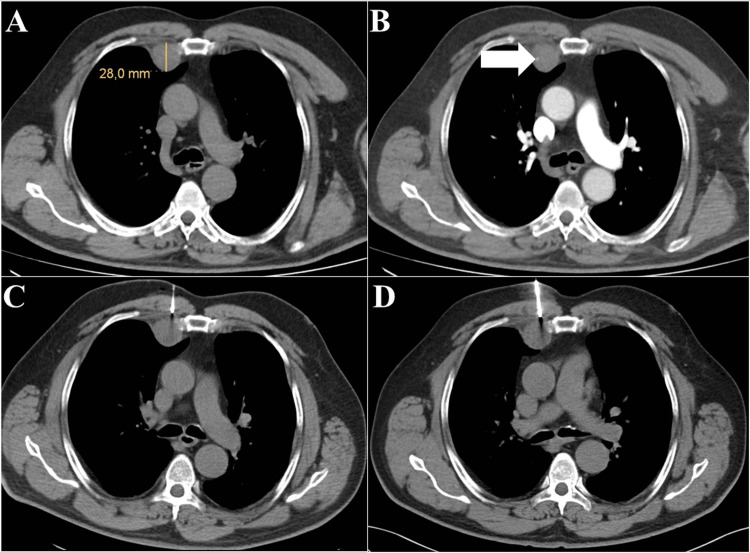
False negative result. Hypovascular focal lesion in the right parasternal space on nonenhanced (A) and contrast-enhanced computed tomography in the arterial phase (B) showing encasement of the internal thoracic artery (arrow). Intraprocedural computed tomography (in the supine position) with two biopsy needle insertions (C, D). Histology revealed connective tissue with atypical cells, but more precise classification was not possible. Surgical excision biopsy confirmed leiomyosarcoma.

Six (4.2%) patients had false-negative final results. In four cases, biopsies revealed necrotic inflammation and normal connective tissue structures, and in the case of two patients, atypical cells were present but were unable to be classified more precisely (Figure 3). However, clinical and imaging suspicion of malignancy remained high. All six patients were referred for surgical resections. Final histological examination confirmed leiomyosarcoma in two cases, and chondrosarcoma, T-cell lymphoma, metastatic breast carcinoma, and metastatic prostate carcinoma in one case each. The biopsy technique was standard in these cases, but the samples were taken from non-viable or marginal regions of the lesions.

**Figure 4:**
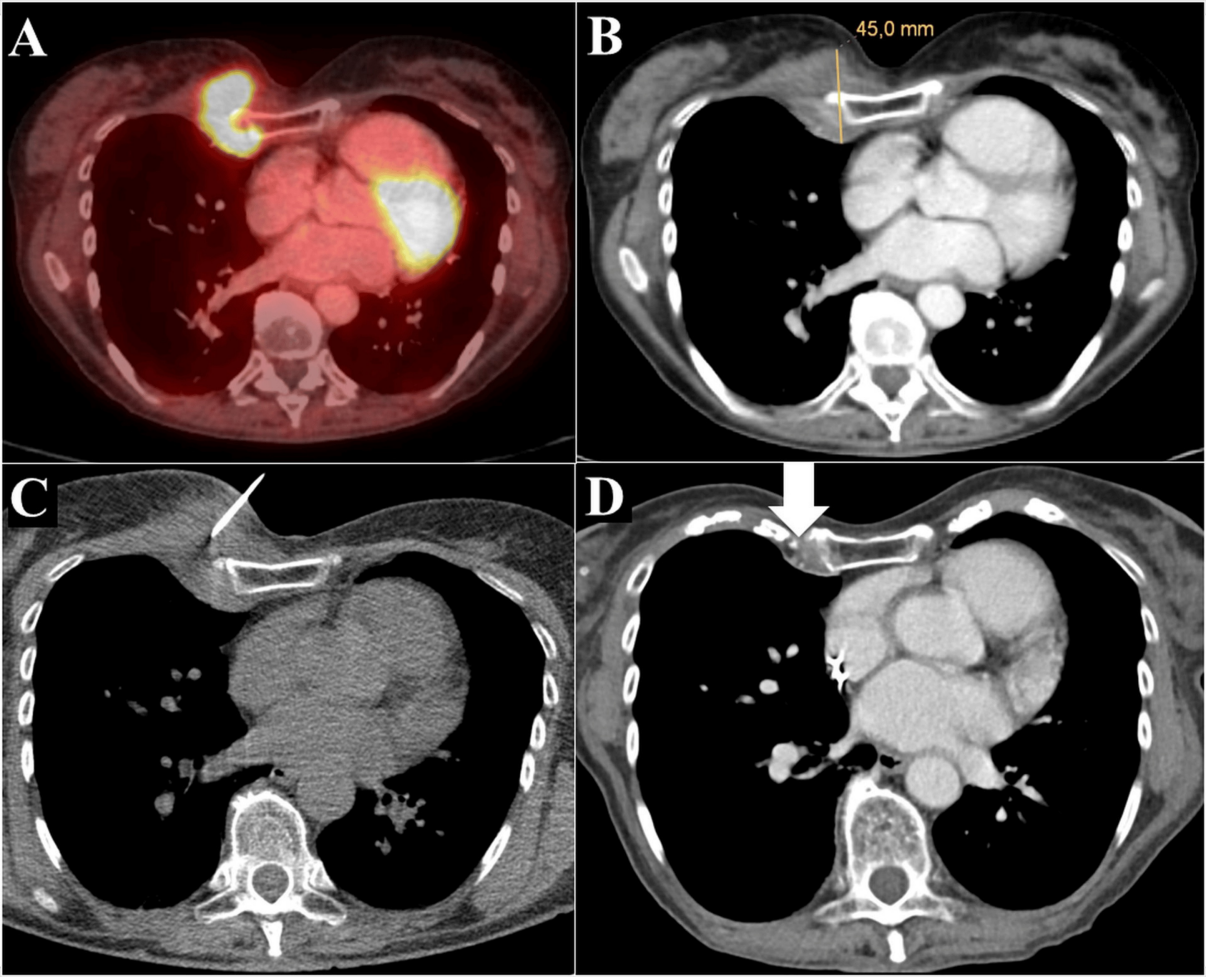
True negative result. Focal lesion in the right parasternal space on positron emission tomography/computed tomography (A) and contrast-enhanced CT in the portal venous phase (B). The placement of the biopsy needle (C, patient in supine position) with a short skin-to-lesion distance.  Definitive histological diagnosis was inflammatory pseudotumor. Follow-up contrast-enhanced CT in the portal venous phase after three months (D) demonstrated complete disappearance of the lesion (arrow).

A thorough long-term follow-up was required for the patients classified as true negative. A total of 16 (11.2%) patients were included in this group. A detailed overview can be found in Table 2. All these cases were validated by follow-up imaging (CT, MRI or PET), showing lesions that remained stable, decreased in size, or completely resolved over at least one year without treatment (Figure 4). Surgical excision was not required for verification of benign diagnoses in this group.

**Table 2:** The number of true negative results.

Diagnosis	Number
Inflammatory pseudotumor	5 (31%)
Necrotic inflammatory tissue	3 (19%)
Rheumatoid nodule	2 (13%)
Extramedular hemopoiesis	2 (13%)
Granulomatous inflammation	1 (6%)
Amyloid	1 (6%)
Elastofibroma	1 (6%)
Angiolipoma	1 (6%)
Number	16 (100%)

**Figure 5:**
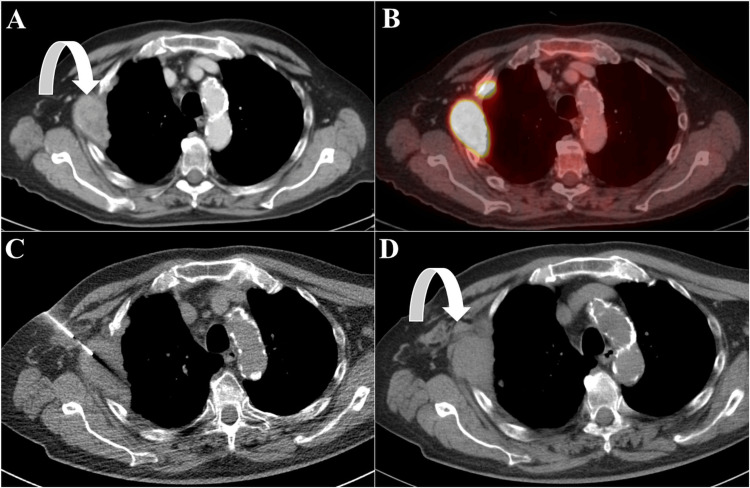
Hemorrhage. Vascularized focal lesion in the right chest wall on contrast-enhanced CT in the portal venous phase (A, curved arrow) and on positron emission tomography/computed tomography (B). Intraprocedural CT showing biopsy needle placement (C, supine position). Hemorrhage in chest wall soft tissue was confirmed (D, curved arrow). The patient was treated conservatively. Definitive histological diagnosis was adenocarcinoma (metastasis of known non-small cell lung carcinoma).

A total of three (2.1%) early complications were observed. All three complications were minor chest wall hemorrhages. Patients were discharged the following day, resulting in a total hospital stay of two days. Histologically, the lesions were metastatic HCC, RCC, and NSCLC (Figure 5). Clinical status remained stable; a conservative approach using analgesia was sufficient. No cases of pneumothorax or pleural cavity bleeding were observed. Blood transfusion, angiography with embolization or surgical revision were not required. No late complications, such as implant metastasis along the biopsy needle track, were observed. The overall complication rate was 2.1%. Statistical analysis showed no significant relationship between complication incidence and final histological results (p = 0.673).

A total of 42 (29.4%) cases were diagnosed as lymphomas. DLBCL was the most frequently diagnosed subtype. It was confirmed as the initial diagnosis in eight cases, as a relapse in five procedures, and four times as a transformation of indolent lymphoma subtypes (CLL/SLL or follicular lymphoma). In cases of suspected lymphoma transformation cases, PET/CT examination was essential for biopsy planning, as the most viable tissue area was targeted for the intervention. A detailed overview of the lymphoma cases is presented in Table 1.

## Discussion

We report our results regarding CT-guided biopsy using a percutaneous approach and its applicability in the diagnosis of focal chest wall lesions. The overall diagnostic accuracy is high; the final result was true positive or true negative in 95.8% of cases. Subtyping was performed by board-certified pathologists when clinically indicated. Metastatic disease of diverse origins was the most frequent histological diagnosis. Primary tumors were verified in 9.1% cases. Using a needle caliber of 16 G or larger and at least two samplings was suitable in the majority of interventions (90.9%). The true negative results were considered the final diagnoses after long-term follow-up or surgical excision. The incidence of complications was 2.1%; all were minor hemorrhages.

Historically, chest wall diseases were verified through clinical examination and plain chest radiography. Surgical excision or resection served as both a diagnostic and a therapeutic option. The final outcome was established only after a non-directed intervention, which often required modification or extension within a relatively short timeframe [12,13]. Tumor evaluation improved primarily with the introduction of ultrasound, CT, PET/CT, and MR into clinical practice. These imaging modalities allow further characterization of tumor origin, definition of its extent and relationships with surrounding structures, and better planning of treatment options [1,8,10]. However, imaging examinations alone often fail to establish a definitive final diagnosis. Histological verification can only be achieved through detailed examination of biopsy specimens [7,14].

For the correct targeting of focal lesions, imaging guidance is absolutely essential. US and CT are the most widely available modalities; in select cases, other imaging techniques (e.g., MR for pediatric indications) may be employed. Advantages of ultrasound guidance include the absence of ionizing radiation and the possibility of real-time procedural control. However, documentation of biopsy images is limited for subsequent postprocedural evaluation [15]. The use of contrast medium (CEUS) may potentially improve diagnostic accuracy, particularly for poorly visible or small focal lesions. This non-invasive procedure provides real-time, detailed information about blood flow and tissue perfusion, which helps in the diagnosis and monitoring of various conditions. CEUS is widely used for evaluating liver lesions, kidney and gallbladder issues, and vascular abnormalities, and its applications are continually expanding into new areas like cancer imaging. A key advantage of CT guidance is verification of the precise biopsy location, which can be demonstrated at each step of the biopsy. Preprocedural imaging can also be compared and fused with intraprocedural scans using artificial intelligence software [16,17]. The use of ionizing radiation is the main shortcoming of CT guidance [18]. For superficially located lesions, biopsy needle holder devices are also available. PET/CT examination can aid in targeting the biopsy needle to the viable areas of large or poorly visualized lesions [19].

In the literature, several authors have presented their results and experience. Overall, objective outcomes remain inconclusive. Particular studies evaluated biopsies using different guidance methods (only ultrasound alone or combined with CT), procedure techniques (fine-needle aspiration (FNA) or core needle biopsy (CNB)), and target structures (focal chest wall lesions alone or grouped with pleural, mediastinal and peripheral lung lesions), some focusing only on primary sarcomas. All involved groups of varying patient numbers [7,14,15,18]. Additional results and experience have also been reported from developing countries [20]. For these reasons, it is not possible to systematically interpret all available information or to acquire universally valid data.

Reported diagnostic accuracy of ultrasound and/or CT-guided FNA or CNB for chest wall pathology varies widely, ranging from 71% to 94.4% [7,15]. Our results correspond to the upper range of published data. Aspiration biopsy and cytology techniques may be useful in lesions with partially fluid characteristics, but they are insufficient for definitive histological diagnostics, including subtyping. Only non-fragmented, compact, and conclusively biopsied samples obtained directly from the tissue of interest are suitable for adequate histological examinations. All of these required characteristics are fulfilled by core-needle biopsy. This procedure is quick, safe, well tolerated by patients, and can be performed using only local anesthesia.

Establishing the correct diagnosis is essential for treatment planning. The majority of cases are managed conservatively. In our cohort, lymphomas, disseminated malignancies and true-negative cases (appropriate for systemic therapy) were confirmed in 86.7% of cases. Surgical resection or excision was appropriate in only a minority of patients.

Only minor complications have been reported in the literature, most commonly pain (at the biopsy site, in the intercostal space or radiating towards the shoulders) and bleeding. Life-threatening hemorrhage has not been reported in the scientific literature. Reported rates of minor hemorrhage range from 2% to 7% [7,15]. The overall complication rate in our cohort was 2.1%. Symptomatic conservative treatment was sufficient in all cases. Needle track seeding of tumorous cells is an extremely rare complication; in lung cancer biopsies extending into the chest wall, the incidence has been reported as less than 0.08% [21]. A slightly higher frequency of seeding was reported in biopsies of pleural mesothelioma [22]. 

Limitations

The limitations of our study include its retrospective data search and analysis; the absence of a control group and its single-center setting. Previously published cohorts have generally been smaller than ours, combined with other lesions than only the chest wall, and the number of interventions assessed in our study is higher than most previously published reported.

## Conclusions

Percutaneous biopsy performed for suspected chest wall malignancy is still not a widely used procedure in plenty of departments. Our results verify metastatic disease (NSCLC as the most common individual diagnosis), lymphomas, and benign histological processes in the majority of cases. In all these cases, systemic therapy is indicated prior to surgical excision or resection. 

CT-guided percutaneous core needle biopsy performed under local anesthesia demonstrates high diagnostic accuracy (more than 95%) for establishing the histological diagnosis of focal chest wall lesions, including precise subtyping. The procedure is rapid, uncomplicated, and well tolerated by patients, making it a reliable option for routine clinical practice.
